# Predicting clinical trial duration via statistical and machine learning models

**DOI:** 10.1016/j.conctc.2025.101473

**Published:** 2025-03-31

**Authors:** Joonhyuk Cho, Qingyang Xu, Chi Heem Wong, Andrew W. Lo

**Affiliations:** a MIT Laboratory for Financial Engineering, Cambridge, MA, USA; b MIT Department of Electrical Engineering and Computer Science, Cambridge, MA, USA; c MIT Computer Science and Artificial Intelligence Laboratory, Cambridge, MA, USA; d MIT Sloan School of Management, Cambridge, MA, USA; e Santa Fe Institute, Santa Fe, NM, USA

**Keywords:** Clinical trial, Cox proportional hazards model, Feature importance, Machine learning, Survival analysis

## Abstract

We apply survival analysis as well as machine learning models to predict the duration of clinical trials using the largest dataset so far constructed in this domain. Neural network-based DeepSurv yields the most accurate predictions and we identify key factors that are most predictive of trial duration. This methodology may help clinical researchers optimize trial designs for expedited testing, and can also reduce the financial risk of drug development, which in turn will lower the cost of funding and increase the amount of capital allocated to this sector.

## 1 Introduction

Despite groundbreaking advances in biomedicine, a significant funding gap remains in financing translational biomedical research, from preclinical animal studies to phase 3 clinical trials, a phenomenon known as the “valley of death” in novel drug development [1]. Among the institutional challenges to bridging this funding gap are the low probability of success [2], the significant capital investment [3], and the long duration of clinical trials [4]. While the low probability of success can be effectively remedied via the “multiple-shots-on-goal” approach of parallel drug discovery [5], the lengthy duration of clinical trials is often necessary to recruit a sufficient number of patients to demonstrate the safety and efficacy of the drug candidate at a target significance level and power. The former study [4] analyzed more than 17,000 trials, finding that the median duration of phase 2 trials increased from 33 months in 2008 to 40 months in 2015, while the median duration of phase 3 trials increased from 33 to 39 months during the same period. For pharmaceutical companies, this long duration decreases the financial value of novel drug development, since it discounts the future revenues of drug sales (should the drug be approved) and increases the capital needed to operate clinical testing sites and perform interim data analyses. Likewise, for patients, the long duration prevents potentially effective therapies from reaching those who are in dire need of care. To address the challenges of a long trial duration, several novel trial designs have been proposed and implemented to expedite the clinical testing process without sacrificing statistical significance or power. Master protocols, including basket trials, umbrella trials, and platform trials, allow the concurrent clinical testing of multiple drug candidates or diseases, often with a shared control arm [6], [7]. For diseases with no effective treatment, patients may be willing to accept a higher risk of adverse effects (i.e., a higher Type I error) in exchange for the expedited approval of an effective treatment (i.e., a lower Type II error). Novel trial designs, based on Bayesian decision analysis, can strike the optimal balance between Type I and II errors for different diseases based on disease severity [8], [9], [10], [11], [12], [13]. For certain infectious diseases, human challenge trials (HCTs) may be employed to expedite the clinical testing of vaccine candidates. A recent simulation analysis [14] revealed that timely initiation and expedited execution of HCTs are critical in preventing a large number of infected cases and deaths from COVID-19. While these unconventional trial designs are best employed under special circumstances, it is also important to systematically analyze the common factors that may affect the duration of all trials and to accurately predict the duration of future trials using these factors. An accurate prediction of a trial’s duration not only facilitates more efficient allocation of capital and resources in clinical testing by pharmaceutical companies, it may also help clinical researchers shorten the trial by optimizing the trial design. To the best of our knowledge, our work is the first to apply both traditional statistical methods and novel machine learning models of survival analysis to predict clinical trial duration, using the largest dataset in this domain.

## 2 Literature review

There is a rich literature on estimating the duration of clinical trials due to their practical importance to pharmaceutical companies. For clinical trials whose primary outcome data is largely uncensored (e.g., COVID-19 infection within 14 days after vaccination), the trial duration is typically estimated using the expected number of patients needed to demonstrate the target significance level and power of the trial, as well as the expected patient enrollment rate [15]. However, for event-based trials whose primary outcome data is censored (e.g., long-term survival or disease progression), the accurate estimation of the survival function (i.e., the probability distribution of the time-to-event) is also essential in predicting the time of interim analysis and trial duration [16]. Early work in this domain used parametric stochastic processes such as the Poisson process and its extensions to model patient enrollment [17], [18]. Bayesian techniques are commonly used to update the probability distribution of trial duration with the observed time-to-event of enrolled patients [15], [19], [20], [21]. Nevertheless, these parametric statistical models impose strong assumptions on the distribution of patient enrollment and the time-to-event. As a result, their prediction accuracy is poor if the model is misspecified [15]. In recent years, given the rapidly growing amount of clinical trial data, machine learning models have been increasingly used to predict patient enrollment. Liu et al. [22] trained machine learning models to predict the time of 50%, 90%, and total enrollment using trial features such as disease indication, trial phase, sponsor, and location. These “bottom-up” approaches found in the literature focus on predicting the enrollment rate. However, they are either tailored to model specific types of trials (e.g., immuno-oncology trials [23]), or do not use sufficient empirical data to validate their prediction [24]. Our work contributes to the literature in two aspects. First, in contrast to the “bottom-up” approach to predict patient enrollment per period, we take a “top-down” approach and directly predict trial duration from a wide variety of trial features, using the Citeline database, which has more than 86,000 trials [25]. In addition, we compare the prediction performance of traditional statistical methods versus machine learning models and identify the key factors that correlate with the duration. To handle ongoing trials whose duration data is right-censored, we apply statistical and machine learning models in the domain of survival analysis. The models used in our analysis are systematically reviewed [26]. Several previous works [27], [28], [29] trained machine learning models using the Citeline database to predict novel drug development outcomes and provide the data query methodology for our work, as described in the next section.

**Table 1:** Clinical trial features extracted from the Citeline database and used to predict trial duration.

Feature	Examples	Type
Drug Origin	Biological, Chemical, Natural Product, etc.	Multi-Label
Drug Medium	Cream, Solution, Tablet, etc.	Multi-Label
Drug Delivery Route	Injectable, Oral, Topical, etc.	Multi-Label
Trial Region	North America, Western Europe, Asia, etc.	Multi-Label
Trial Tag	Biomarker/Efficacy, Expanded Indication, First in Human, etc.	Multi-Label
Trial Phase	1, 1/2, 2, 2/3, 3	Multi-Label
Trial Target Accrual	Number of target accrual	Numeric
Trial Patient Min Age	Minimum age for patient	Numeric
Trial Patient Max Age	Maximum age for patient	Numeric
Trial Patient Age Range	Difference between maximum and minimum ages for patient	Numeric
Trial Patient Age Group	Adults, Children, Older Adults	Multi-Label
Trial Patient Gender	Both, Female, Male	Multi-Label
Trial Endpoint	Survival, Disease Progression, Pharmacokinetics, etc.	Multi-Label
Therapeutic Area	Oncology, Cardiovascular, Vaccines, etc.	Multi-Label
Sponsor Type	Academic, Government, Industry (Top 20 Pharma), etc.	Multi-Label
Disease Prevalence	Prevalence of target disease	Numeric
Disease DALY (Avg)	Average DALY of target disease	Numeric
Disease DALY (Max)	Maximum DALY of target disease	Numeric
Trial Start from Drug Launch	Years between a drug’s market launch and the trial start; −1 indicates the drug has not yet launched.	Numeric
Never Developed	Binary indicator; 1 if the drug is not yet launched at the time of the trial, 0 if it is already on the market.	Binary
Trial Start Date	Date of trial start	Numeric

**Table 2:** Summary of clinical trial durations from the Citeline dataset (in months). Panel (a) provides overall statistics by phase, while Panel (b) presents a breakdown by therapeutic area for phases 1, 2, and 3. For both panels, *Trials* indicates the total number of trials in that category, and *Drugs* indicates the number of unique drugs. *Mean* and *SD* refer to the arithmetic mean and standard deviation of trial duration, respectively, while *25% Qt.*, *Median*, and *75% Qt.* describe distribution quartiles.

(a) Overall Duration by Phase. This panel shows the aggregated trial data across phases 1, 1/2, 2, 2/3, and 3. The columns under ‘‘Duration’’ provide summary statistics (mean, SD, and quartiles) for the length of each phase.							

Phase	Trials	Drugs	Duration Mean	Duration SD	Duration 25% Qt.	Duration Median	Duration 75% Qt.

1	22,129	11,500	16.0	21.1	2.7	7.5	21.0
1/2	6,141	3,876	36.2	27.6	15.5	30.0	50.0
2	34,053	9,854	30.2	25.6	12.3	23.3	40.3
2/3	3,371	2,195	29.3	26.5	11.1	22.1	39.0
3	24,672	6,804	30.2	27.3	12.6	22.5	38.9

Total	90,366	20,760	27.1	26.1	9.0	19.8	37.0

(b) Duration by Therapeutic Area (Phases 1, 2, and 3). Each row represents a different disease category, showing how trial durations can vary by therapeutic focus.						

Therapeutic area	P1 Num.	P1 Mean (SD)	P2 Num.	P2 Mean (SD)	P3 Num.	P3 Mean (SD)

Oncology	3,993	39.4 (27.7)	11,309	45.2 (29.2)	3,533	58.6 (36.4)
Cardiovascular	2,240	9.0 (14.5)	2,437	25.9 (21.0)	2,862	30.5 (24.0)
CNS	4,761	11.2 (15.9)	6,681	25.0 (20.2)	5,545	26.9 (20.6)
Metabolic/Endocrinology	3,902	9.0 (13.7)	3,695	24.4 (22.6)	3,941	26.4 (28.1)
Genitourinary	509	9.3 (14.4)	693	22.3 (17.7)	775	24.9 (17.3)
Autoimmune/Inflammation	3,454	11.9 (16.3)	5,592	22.8 (20.1)	4,331	24.7 (20.0)
Ophthalmology	359	17.2 (18.2)	950	20.4 (16.9)	741	24.5 (18.8)
Infectious Disease	3,750	12.1 (14.5)	4,560	19.6 (18.0)	4,091	22.1 (20.0)
Vaccines	1,159	16.9 (14.7)	1,112	16.5 (16.5)	1,466	14.8 (15.9)

## 3 Data and methods

### 3.1 Data query and preprocessing

We query the historical clinical trial data from the Citeline database [25], one of the largest datasets in this domain. Detailed descriptions of trial features are summarized in Table 1. These features are either categorical or numerical, and for multi-labeled categorical features with k categories (e.g., a drug developer may conduct clinical trials in k different countries), we apply one-hot encoding to generate k binary variables. In our analysis, we focus exclusively on features that are available before the trial starts. The features can be classified into three groups: trial-related, drug-related, and disease-related. For drug-related data, we link the trial information (from Trialtrove) with the corresponding drug dataset (Pharmaprojects) from Citeline. For disease-related data, we match the disease information to the Global Burden of Disease dataset [30] to obtain measures of prevalence and severity. In this work, disease severity is quantified using disability-adjusted life years (DALY), which combine years of life lost due to premature mortality with years lived with disability to provide a comprehensive measure of disease burden.

To preprocess the raw data for the machine learning models, we first exclude trials with unknown start dates. When available, we use the clearly reported trial duration as the duration measure. For trials lacking a reported duration but with clear start and end dates (trial primary completion dates), we compute the duration as the difference between the end and start dates (in months). For trials with a known start date but lacking both a reported duration and an end date, we treat the trial duration as right-censored. Our final dataset comprises 90,366 clinical trials involving 20,760 drugs, making it the largest reported in the literature. Detailed summary statistics are shown in Table 2(a), and more detailed statistics by therapeutic area are provided in Table 2(b). Note that the total trial count in Table 2(a) is lower than the sum in Table 2(b) because some trials are classified under multiple therapeutic areas.

Due to different standards in the post-study reporting of clinical trial results (especially before the 2007 FDA Amendments Act), there is considerable missingness in certain clinical trial features, which we impute via median imputation [27].

### 3.2 Traditional survival analysis

Here we introduce the notation used in the rest of the paper. Let $T_{i}>0$ denote the duration of the ith clinical trial in the dataset and $X_{i}$ denote its d-dimensional feature vector. The survival function, S(t)=P(T>t), is the probability that the duration is longer than t. Under mild regularity conditions on S(t), a useful and mathematically equivalent way to characterize the survival function S(t) is through its hazard function h(t), defined by

(1) $$h\left(t\right)=\underset{\Delta t\to 0}{\lim}\frac{P\left(t\leq T<t+\Delta t\;|\;T>t\right)}{\Delta t}=-\frac{S^{\prime }\left(t\right)}{S\left(t\right)}$$

Note that h(t)≥0, since S(t) monotonically decreases from 1 to 0 as t increases from 0 to ∞. Since the duration of currently ongoing trials is right-censored (i.e., observed in the future of our analysis), we use the binary variable $\delta_{i}$ to denote whether the duration, $T_{i}$, of the ith clinical trial is right-censored ($\delta_{i}=1$) or observed without censoring ($\delta_{i}=0$). We use non-parametric (Kaplan–Meier), semi-parametric (Cox regression), and parametric (accelerated failure time) statistical models of survival analysis to estimate the survival function, S(t), from empirical data and predict the trial duration through the median time, $t_{1/2}$, of S(t). We use the models implemented in the **scikit-survival v 0.17.1** library (Cox regression) [31] and **lifelines** library (Weibull AFT) of Python 3.8 [32].

### 3.3 Machine learning models

#### 3.3.1 Models based on decision trees

The decision tree is a commonly used machine learning model that is simple to train and easy to interpret [33]. During the training stage, each tree node is split into subsequent child nodes by maximizing the homogeneity of data samples within each child node. Common metrics of homogeneity include the mean-squared error (for regression analysis) and entropy (for classification analysis). We use the decision tree models implemented in the **scikit-survival v 0.17.1** library of Python 3.8 [31].

#### Survival tree

To train the survival tree on the training dataset, each intermediary tree node is split into its child nodes by maximizing the value of the log-rank test [34]. For each terminal leaf node n, a Kaplan–Meier survival function $S_{n}\left(t\right)$ is computed using the durations of clinical trials in this node. To evaluate the prediction performance during the testing stage, the survival function, $S_{n}\left(t\right)$, is used to predict the duration of a new clinical trial, which is assigned to node n by applying the partition rules on its features $X_{i}$. To prevent overfitting of the training dataset, we choose the following regularization hyperparameters: maximum depth of the tree, $d_{max}=10$; and minimum samples of each split, $n_{split}=50$, after tuning the hyperparameter values.

#### Random survival forest

Although the survival tree is simple to train and highly interpretable, its structure is unstable and sensitive to the distribution of data samples in the training dataset. The random survival forest effectively reduces the variances of the survival tree model via the “bagging” approach, i.e., by training multiple survival trees, each with its training data bootstrapped from the original dataset [35]. To predict the duration of a clinical trial in the testing dataset, the predictions of all survival trees in the forest are averaged. We choose the following hyperparameter values: the number of survival trees, $n_{tree}=100$; the minimum samples of each split, $n_{split}=20$; and the minimum sample per terminal leaf, $n_{leaf}=20$ with maximum depth $d_{max}=3$. We make each split during training using the splitting ratio, $r_{split}=50\text{\%}$, of the total number of features.

#### Gradient boosting survival trees

The gradient boosting tree method improves simple decision trees via the technique of boosting [36], i.e., iteratively reducing the residual prediction error of previously trained trees by adding a new tree into the ensemble. Gradient boosting survival trees apply the same technique to survival trees [37]. We train a gradient boosting survival tree that minimizes the partial likelihood loss of Cox’s proportional hazards model:

(2) $$L_{GBST}=\overset{n}{\underset{i=1}{\sum }}\left(1-\delta_{i}\right)\left[f\left(X_{i}\right)-\log\left(\underset{j\in R_{i}}{\sum }\exp(f\left(X_{i}\right))\right)\right],$$

where $f\left(X_{i}\right)$ denotes the hazard function, which is the weighted average of the outputs from all decision trees in the gradient boosting ensemble, and $R_{i}$ denotes the set of data samples whose duration is longer than $T_{i}$. We choose the following hyperparameter values: the number of estimators, $n_{est}=100$; a maximum depth of each tree, $d_{max}=3$; a minimum sample per terminal leaf, $n_{leaf}=100$; and the learning rate, $\alpha =10^{-3}$.

#### 3.3.2 Models based on neural networks

Deep learning models based on neural networks have been the main driving force behind the artificial intelligence revolution in the past decade, outperforming traditional machine learning models in domains such as computer vision, natural language processing, and reinforcement learning [38], [39], [40]. In recent years, neural networks have also been applied to survival analysis [26]. We train two neural network models as implemented in the **PyCox** library of Python 3.8.

#### DeepSurv

DeepSurv is the nonlinear generalization of the traditional Cox proportional hazard model [41]. Instead of using a linear function, $\beta^{T}X_{i}$, in the exponent of the hazard function, DeepSurv uses a nonlinear function, $h_{\theta }\left(X_{i}\right)$, which takes the functional form of a feedforward neural network parameterized by θ. This generalization greatly increases the model’s capacity to model the nonlinear impact of trial features $X_{i}$ on the trial duration. We train a neural network $h_{\theta }\left(x\right)$ with two hidden layers of dimensions 200 and 100, respectively. We choose ReLU as the activation function [42] and optimize the model parameters via the Adam algorithm [43], with a batch size of 256, training epochs of 300, a dropout rate of 0.1, and a learning rate, $\alpha =5\times 10^{-3}$.

#### Neural multi-task logistic regression

Similar to DeepSurv, the neural multi-task logistic regression is a nonlinear generalization of the traditional multi-task logistic regression (MTLR) model for survival analysis [44]. Traditional MTLR first partitions the future time period into N intervals, $0=t_{0}<t_{1}<\cdots <t_{N-1}<t_{N}=T_{max}$, and uses a logistic regression on trial features $X_{i}$ to predict the probability, $p_{n}=P\left(t_{n-1}\leq T_{i}<t_{n}|X_{i}\right)$, for all 1≤n≤N. Since it performs a separate logistic regression for each time interval, it bypasses the strong proportional hazard assumption of the Cox model. The neural MTLR replaces the linear logit in logistic regression with a nonlinear feed-forward neural network to compute $p_{n}$. For a given partition of time intervals, the survival function, S(t), is piecewise constant in each interval $\left[t_{n-1},t_{n}\right)$, and is given by $S\left(t\right)=\sum_{n=1}^{N}p_{n}I\left\{t<t_{n}\right\}$. This is an effective approximation of the true survival function when the interval lengths, $t_{n}-t_{n-1}$, are sufficiently small. We choose the number of partitions, N=100 time intervals. The other model hyperparameters are the same as for DeepSurv.

#### 3.3.3 Survival support vector machine

The survival support vector machine (SSVM) method is a ranking algorithm that predicts the relative order of durations (i.e., the binary outcome, $I\left\{T_{i}>T_{j}\right\}$) for a pair of clinical trials, i and j, rather than predicting their actual durations, $T_{i}$ and $T_{j}$, individually [45]. If both $T_{i}$ and $T_{j}$ are right-censored, their ranking cannot be determined. However, if at least one of $T_{i}$ and $T_{j}$ is observed (e.g., $\delta_{j}=0$), their ranking can be determined if one of the two conditions holds: (1) $T_{i}$ is also observed, or (2) $T_{i}$ is right-censored but $T_{i}>T_{j}$. Formally, the pairs of clinical trials whose durations may be ranked are:

(3) $$P=\left\{\left(i,j\right)\;|\;\left(T_{i}>T_{j}\right)\wedge \left(\delta_{j}=0\right),\;\text{where}\;1\leq i,j\leq n\right\}.$$

We then train SSVM by minimizing the loss function:

(4) $$L_{SSVM}=\underset{w}{\min}\frac{\gamma }{2}{\left\|w\right\|}^{2}+\underset{\left(i,j\right)\in P}{\sum }\max\left\{0,1-w^{T}\left(X_{i}-X_{j}\right)\right\},$$

where w consists of the model parameters learned from the data, and γ is the $L_{2}$ regularization factor (which we set to γ=0.001). The prediction performance is measured by Harrell’s concordance index [46], which is the ratio of the number of correctly ranked pairs of trial durations to all comparable pairs, |P|. We use the SSVM model implemented in **scikit-survival v 0.17.1** library of Python 3.8 [31].

### 3.4 Measurement of prediction performance

We measure the prediction accuracy using the concordance index (c-index) and compare the accuracy of traditional survival models to machine learning models. Five-fold cross-validation is used on the split training and testing datasets to find the best hyperparameters for each model. The performance of each model is then calculated by averaging the c-indices of five independent splits of training and testing data.

**Fig. 1:**
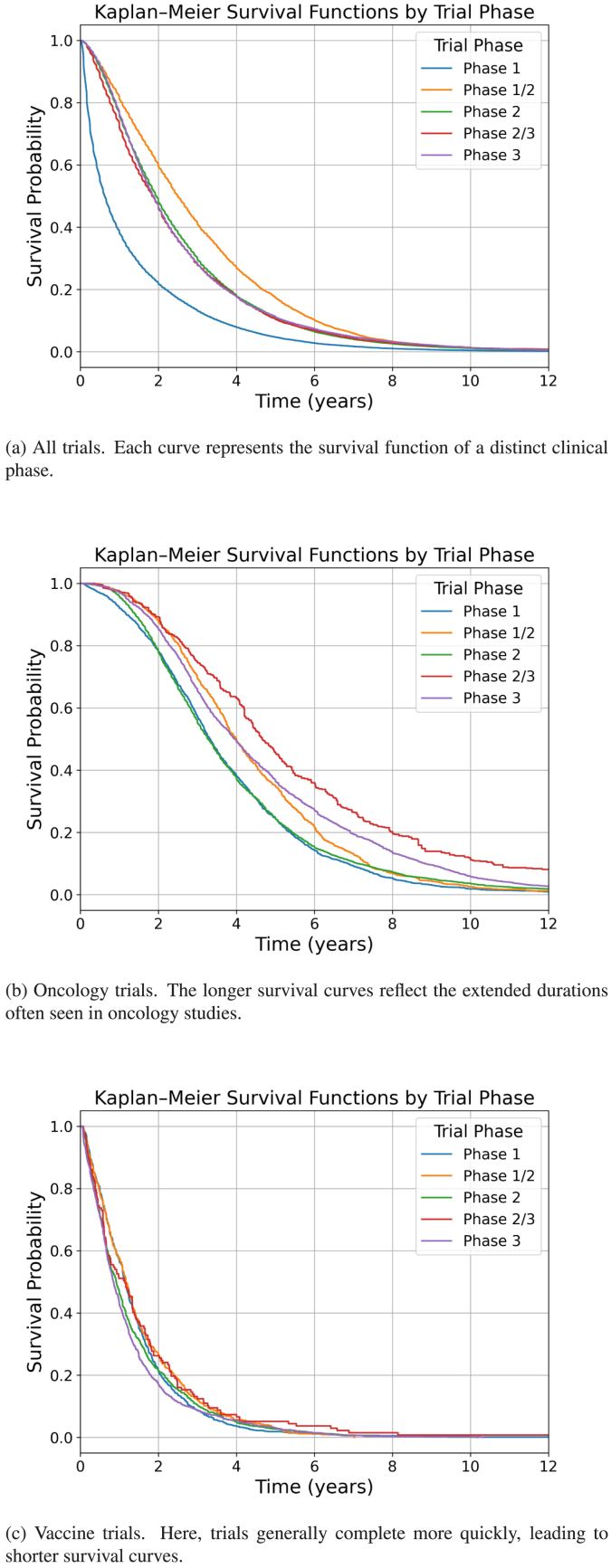
Kaplan–Meier survival functions of trial durations by clinical phase, subdivided by disease category. Panel (a) shows all trials, (b) focuses on oncology trials, and (c) highlights vaccine trials. The data illustrate how different therapeutic areas can influence overall trial length, with oncology trials tending to last longer and vaccine trials often completing more quickly.

**Fig. 2:**
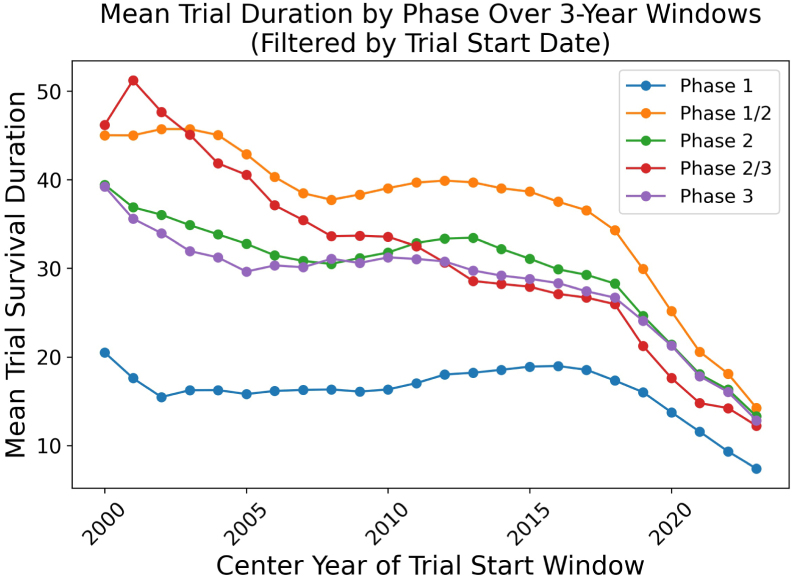
Mean trial duration by phase over 3-year rolling windows, filtered by trial start date. Each data point corresponds to a center year for which we include trials that started up to 1 year before and after. For example, a center year of 2020 captures trials initiated between January 1st, 2019 and December 31th, 2021. This rolling-window approach smooths year-to-year fluctuations and reveals an overall decline in mean trial duration over the past two decades. However, the steep drop in the most recent 3–4 years is partly driven by selection bias, as many longer-running trials initiated during that time are still ongoing (i.e., censored) in our dataset.

## 4 Results

### 4.1 Non-parametric analysis and overall statistics

The summary statistics of clinical trial duration by phase are presented in Table 2(a), and the corresponding Kaplan–Meier survival functions are shown in Fig. 1. In panel (a), we observe the overall dataset, whereas panels (b) and (c) focus on oncology and vaccine trials, respectively. Overall, phase 1 trials exhibit the shortest durations, while Phase 1/2 trials tend to be the longest. By contrast, phases 2, 2/3, and 3 have more comparable duration distributions.

As shown in Table 2(b), a key factor behind the similar durations for phase 2 and phase 3 is the differing composition of disease categories. Oncology trials (which generally last longer) constitute a larger proportion of Phase 2 but often do not progress to phase 3, reducing the share of long oncology trials at later stages. Conversely, some diseases with shorter trials (e.g., CNS, Metabolic/Endocrinology, Vaccines) are more common in phase 3, further narrowing the overall duration gap. This pattern is reflected in Fig. 1(b), where oncology trials generally show longer survival curves, and in Fig. 1(c), where vaccine trials tend to have shorter durations.

Additionally, Table 2(b) indicates that within the same disease category, trial durations generally increase with later phases, reflecting the more extensive and complex nature of advanced trials. By splitting Fig. 1 into subfigures, we highlight these disease-specific trends and demonstrate how certain therapeutic areas can substantially influence the observed durations at each clinical phase.

Beyond comparing phases, we also examine the temporal evolution of clinical trial durations, as shown in Fig. 2. Over the past two decades, several initiatives and methodological improvements—such as adaptive trial designs [47] and the accelerated approval pathway [48]—have been introduced to enhance the speed of clinical research. These efforts partly explain the overall downward trend in mean trial duration. However, the marked decline in the most recent few years should be interpreted cautiously, as many of the longer-running trials that began in that period remain ongoing and are therefore censored in our dataset, artificially reducing observed trial lengths.

### 4.2 Prediction performance

Table 3 presents the c-index of each statistical and machine learning model across five independent training–testing splits. Overall, the DeepSurv model achieves the highest mean c-index of 0.777 (with a standard error of 0.002), outperforming both classical approaches (Cox, Weibull AFT) and other machine learning models. Random forest also shows competitive performance, underscoring the effectiveness of ensemble tree-based methods for survival prediction. Meanwhile, neural MTLR and SSVM achieve mean c-indices above 0.750. In contrast, the single survival tree attains the lowest c-index (0.740), possibly due to overfitting. These results suggest that neural-based survival models (DeepSurv, neural MTLR) and well-tuned ensembles (random forest) can effectively capture the complex relationships in clinical trial data, while simpler or more traditional methods lag slightly behind.

### 4.3 Feature importance

#### 4.3.1 Correlation coefficient

We compute Spearman’s and Pearson’s correlation coefficients as direct measures of feature importance in predicting trial duration. Because our dataset includes binary features and may exhibit non-linear relationships, Spearman’s correlation is particularly suitable for capturing monotonic associations [49]. Table 4 shows both correlation measures, sorted by the magnitude of Spearman’s coefficient. Notably, “Therapeutic Area: Oncology” has a strong positive coefficient, reflecting the extended time frames often required for oncology studies. By contrast, phase 1 trials show a high negative coefficient, underscoring their relatively shorter durations. Moreover, endpoints play a critical role: trials with endpoints such as survival, which demand long-term follow-up, tend to exhibit higher positive correlations with duration. Although the Pearson correlation coefficients differ slightly in value, they follow a similar trend, reinforcing the overall associations observed between these features and trial duration. For complete results and additional details regarding the correlation coefficient, please refer to **Supplementary File S1**.

**Table 3:** Prediction performance (measured by c-index) of statistical and machine learning models. We train each model using 5 independent splits of training and testing datasets and report the mean and the standard error of the c-index. Gradient boosting trees (colored in red) shows the best prediction performance.

Model	Split 1	Split 2	Split 3	Split 4	Split 5	c-index (mean)	c-index (SE)
Cox Regression	0.756	0.754	0.751	0.753	0.758	0.754	0.003
Weibull AFT	0.756	0.754	0.751	0.752	0.757	0.754	0.003
Survival Tree	0.741	0.741	0.735	0.743	0.741	0.740	0.003
Random Forest	0.763	0.764	0.758	0.761	0.766	0.762	0.003
Gradient Boosting	0.745	0.745	0.740	0.745	0.747	0.744	0.003
							
Neural MTLR	0.740	0.781	0.742	0.746	0.749	0.752	0.015
SSVM	0.759	0.758	0.755	0.743	0.741	0.751	0.008

**Table 4:**
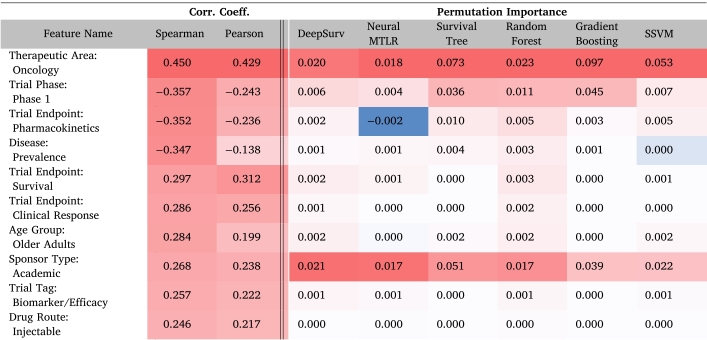
Top 10 features selected by absolute Spearman correlation coefficient to trial duration, showing both Spearman and Pearson correlation coefficients, followed by permutation importance for each ML-based survival model. Within each model’s columns, darker red indicates higher importance (or correlation coefficient) among these features, while blue marks lower importance.

#### 4.3.2 Permutation importance

Permutation importance is a generic method to measure the importance of each feature on the prediction performance of any machine learning model [50]. For a given feature, the permutation importance is defined as the decrease in prediction performance if the values of this feature are randomly permuted across the samples in the testing dataset, while the values of all other features are fixed.

Table 4 shows the permutation importance of the top 10 trial features with the largest magnitudes of Spearman’s correlation coefficient with the trial duration. Some features such as “Therapeutic Area: Oncology” and “Sponsor Type: Academic” exhibit consistently high permutation importance across all models, in line with their strong correlation coefficients. However, other features do not attain equally high permutation importance despite relatively large correlations. This discrepancy often arises when the information conveyed by these features overlaps with that of other correlated variables, or when a feature’s binary nature results in too few positive (or negative) instances to strongly influence the model.

To analyze the feature importance of individual models, we list the top 10 most important features for each model in Table 5. The color scheme groups the features by category (e.g., therapeutic area, sponsor type, trial phase), making it easier to visually distinguish different types of features. Despite differences in prediction performance, the models consistently identify a similar set of features as having the greatest impact on trial duration. In particular, whether the drug treats an oncology indication remains the top feature across all models, with the sponsor type (academic or government), the start date of the trial, and the trial phase (phase 1) also ranking highly. This consistency underscores the robust influence of these features on trial duration. For complete results and additional details regarding the permutation importance, please refer to **Supplementary File S1**.

**Table 5:** Top 10 features with the highest permutation importance for each machine learning model (listed in descending order). Each cell is color-coded according to the feature category (e.g., therapeutic area, sponsor type, trial region, numeric feature) to make the different types of features more visually distinguishable.

## 5 Discussion

By applying different types of survival analysis models to predict clinical trial duration using the largest dataset to date in this domain, we systematically identify several key factors that influence trial duration. The most important factor, as measured by both correlation coefficient and permutation importance, is whether the drug treats an oncology indication. The long duration of oncology trials is partially due to the necessity of post-treatment follow-ups with trial participants over an extended period in order to measure trial endpoints such as long-term patient survival and disease progression. Though an extended follow-up period is often required to discover statistically significant clinical efficacy, the overall duration of oncology trials can be shortened through the wider application of novel trial designs such as platform trials [6] and Bayesian trials [8], whose target significance level and duration can be tailored according to the prevalence and severity of different diseases.

In addition, clinical trials conducted by academic medical centers (Spearman coefficient: 0.268) and government agencies (Spearman coefficient: 0.241) are significantly longer than those conducted by pharmaceutical companies (Spearman coefficient: −0.145). This difference indicates a need for greater public–private partnerships in novel drug development, especially for rare diseases, which do not generate large revenues for pharmaceutical companies. If pharmaceutical companies are given greater financial or regulatory incentives to develop drugs for rare diseases (e.g., in the form of priority review vouchers), the duration of these trials may be significantly shortened for the benefit of those patients in need.

## 6 Conclusion

We apply statistical and machine learning models to predict the duration of clinical trials using the largest dataset constructed for this domain to date. We find that of the models employed, DeepSurv achieves the best prediction performance. Key factors that influence trial duration found across our models include whether the drug treats an oncology indication, the type of sponsor of the clinical trial, the trial phase, target accrual, and the trial region. Our results call for the wider use of novel trial designs and greater public–private partnerships to shorten the typical clinical trial duration.

## CRediT authorship contribution statement

**Joonhyuk Cho:** Writing – original draft, Visualization, Software, Methodology, Investigation, Formal analysis, Data curation, Conceptualization. **Qingyang Xu:** Writing – original draft, Software, Methodology, Data curation, Conceptualization. **Chi Heem Wong:** Writing – review & editing, Validation, Software, Data curation, Conceptualization. **Andrew W. Lo:** Writing – review & editing, Validation, Supervision, Resources, Investigation, Conceptualization.

## Declaration of competing interest

The authors declare the following financial interests/personal relationships which may be considered as potential competing interests: No conflicts of interest are declared for Chi Heem Wong. Joonhyuk Cho and Qingyang Xu report personal investments in publicly traded pharmaceutical companies. Andrew W. Lo reports personal investments in private biotechnology companies, biotechnology venture capital funds, and mutual funds; is a co-founder and principal of QLS Advisors LLC, a healthcare investments advisor, and QLS Technologies LLC, a healthcare analytics and consulting company; is a director of AbCellera, Annual Reviews, BridgeBio Pharma, n-Lorem, Uncommon Cures, and Vesalius Therapeutics; is an advisor to AACR Oncology Development Fund, Apricity Health, Aracari Bio, BrightEdge Impact Fund, Gondola Bio, Health at Scale, Khora Therapeutics, MIT Proto Ventures, Quantile Health, Ride Therapeutics, Roivant Social Ventures, Swiss Finance Institute, Thalēs, Think Therapeutics, and xCures; and, during the most recent six-year period, has received speaking/consulting fees, honoraria, or other forms of compensation from AbCellera, AlphaSimplex Group, Annual Reviews, Apricity Health, Aracari Bio, Atomwise, Bernstein Fabozzi Jacobs Levy Award, BridgeBio, CME, Enable Medicine, Journal of Investment Management, Lazard, MIT, New Frontier Advisors/Markowitz Award, Oppenheimer, Princeton University Press, Q Group, QLS Advisors, Quantile Health, Roivant Sciences, SalioGen Therapeutics, Swiss Finance Institute, Think Therapeutics, Vesalius, and WW Norton.

## Data Availability

The authors do not have permission to share data.

## Appendix A Supplementary data

Supplementary File S1: Full Results (CSV). This file includes the c-index results, correlation coefficients, and permutation importance metrics for all models applied and every feature investigated in our study.

MMC S1 Includes c-index results, correlation coefficients, and permutation importance metrics for all models and every feature investigated.
